# The Invisible Burden: Diagnosing and Combatting Typhoid Fever in Asia and Africa

**DOI:** 10.1093/cid/ciz611

**Published:** 2019-10-15

**Authors:** Virginia E Pitzer, James Meiring, Frederick P Martineau, Conall H Watson, Gagandeep Kang, Buddha Basnyat, Stephen Baker

**Affiliations:** 1 Department of Epidemiology of Microbial Diseases, Yale School of Public Health, Yale University, New Haven, Connecticut; 2 Oxford Vaccine Group, Department of Paediatrics, University of Oxford, and the National Institute for Health Research Oxford Biomedical Research Centre, United Kingdom; 3 Department of Global Health and Development and, United Kingdom; 4 Department of Infectious Disease Epidemiology, London School of Hygiene and Tropical Medicine, United Kingdom; 5 Centre for Tropical Medicine and Global Health, Nuffield Department of Medicine, University of Oxford, United Kingdom; 6 Translational Health Sciences Technology Institute, Faridabad, Haryana, India; 7 Oxford University Clinical Research Unit, Patan Academy of Health Sciences, Kathmandu, Nepal; 8 Hospital for Tropical Diseases, Wellcome Trust Major Overseas Programme, Oxford University Clinical Research Unit, Ho Chi Minh City, Vietnam; 9 Department of Medicine, University of Cambridge, United Kingdom

**Keywords:** enteric fever, *Salmonella Typhi*, paratyphoid, surveillance, blood culture

## Abstract

Measuring the burden of typhoid fever and developing effective strategies to reduce it require a surveillance infrastructure that is currently lacking in many endemic countries. Recent efforts and partnerships between local and international researchers have helped to provide new data on the incidence and control of typhoid in parts of Asia and Africa. Here, we highlight examples from India, Nepal, Vietnam, Fiji, Sierra Leone, and Malawi that summarize past and present experiences with the diagnosis, treatment, and prevention of typhoid fever in different locations with endemic disease. While there is no validated road map for the elimination of typhoid, the lessons learned in studying the epidemiology and control of typhoid in these settings can provide insights to guide future disease control efforts.

Typhoid fever has been recognized as a major cause of morbidity and mortality in humans for centuries. However, the global burden of typhoid fever through human history is uncertain and has only been determined in a limited number of locations [1–3]. The symptoms of typhoid fever, caused by ingestion of the bacterium *Salmonella enterica* serovar Typhi (*S.* Typhi), include prolonged fever, abdominal discomfort, and malaise, which can be serious and progress to include complications such as intestinal perforation [4]. Typhoid fever is considered a disease of poverty and underdevelopment, largely driven by a lack of access to clean water and poor sanitation. Following the introduction of filtration and chlorination of water supplies and wide-scale construction of sewer systems in the United States and western Europe in the early 20th century, typhoid fever was all but eliminated from these countries. Consequently, the overwhelming burden of typhoid fever currently falls disproportionately in low- and lower-middle-income countries (LMICs), including parts of the world where improvements in water and sanitation infrastructure have been slow to materialize or unable to keep pace with the growth of informal urban settlements.

Estimating the burden of typhoid fever (and developing strategies to reduce it) requires carefully conducted surveillance. Several surveillance programs have recently been established across Asia and Africa. These studies, which have attempted to harmonize study methods and use standardized blood culture methods for typhoid diagnosis, have identified high rates of disease (unpublished data). Vaccines potentially capable of inducing effective and long-lasting immunity against typhoid are now available [5]. Policy makers must consider use of these vaccines to complement existing control measures, including the use of appropriate antimicrobial therapy combined with improvements in sanitation, food, and water safety, to ensure that the burden of typhoid disease continues on a downward trajectory.

Here, we present a review of past and current experiences of diagnosing and controlling typhoid across geographically representative surveillance sites in Asia and Africa. We draw on specific examples from India, Nepal, Vietnam, Fiji, Sierra Leone, and Malawi to highlight how collaborations between researchers, local clinicians, and public health officials have helped to assess the impact of typhoid fever. We also describe recent successes and setbacks in developing strategies to reduce the burden of disease in these populations (summarized in Table 1).

**Table 1.** Successes and Challenges in Typhoid Fever Surveillance and Control in 6 Countries from Asia and Africa

Country	Population in 2019^a^	Incidence of Typhoid Fever, per 100 000 Person-Years^b^	Successes in Typhoid Fever Surveillance and Control	Challenges Encountered With Typhoid Fever Surveillance and Control
India	1.37 billion	81–499	Multiple hospital-based, laboratory-based, and population-based active surveillance studies have been conducted since the 1990s	Widespread availability of over-the-counter antimicrobials, leading to lower rates of formal healthcare-seeking, poor sensitivity of blood culture diagnosis, and selection pressure for the emergence of antimicrobial resistance
Nepal	28.6 million	113–436	Conducted randomized controlled trials of treatment options and Vi-polysaccharide and Vi-conjugate vaccines	Other circulating pathogens that cause clinically indistinguishable disease (eg, typhus) lead to common misdiagnoses
				Emergence of antimicrobial resistance
Vietnam	96.5 million	60–149	Economic reform led to improvements in infrastructure and decline in typhoid incidence	It is difficult to estimate the role of any single intervention in leading to the decline in typhoid fever incidence
			Vi-polysaccharide vaccination campaigns were initiated and the first randomized controlled trial of Vi-conjugate vaccine was conducted	
Fiji	890 000	21–100	Recent case-control and serological studies have identified risk factors for infection and potential differences in reporting by age and among iTaukei and Indo-Fijians Vi-polysaccharide vaccination campaign mounted in response to Cyclone Tomas Good antimicrobial stewardship	Typhoid fever cases increased between 2004 and 2008, which may be due to better reporting Higher rates of typhoid fever among iTaukei may reflect more severe pathology of disease in indigenous Fijians
Sierra Leone	7.81 million	194–925	Major investment in the Integrated Disease Surveillance and Response program following the 2014–2016 Ebola epidemic	Laboratory facilities necessary for blood culture testing are lacking
				Most typhoid fever cases diagnosed using the Widal test (which has poor specificity) and/or based on clinical symptoms only
				Financial barriers to effective treatment due to limited antimicrobial supply
Malawi	18.6 million	90–298	Consistent hospital-based blood culture surveillance conducted since 1998 at Queen Elizabeth Central Hospital in Blantyre	More than 10-fold increase in the incidence of typhoid fever following the emergence of the H58 haplotype and associated antimicrobial resistance in 2011
			First country in Africa to conduct a randomized controlled trial of Vi-conjugate vaccine	

^a^Source: United Nations World Population Prospects database (https://population.un.org/wpp/).

^b^The range of the mean typhoid incidence estimate from 3 recent studies [1–3] is presented.

## Typhoid in Asia: India

South Asia has a long history of typhoid fever, which has been well documented among the more than 1 billion people who lived in India before and after colonial times. The British military detailed typhoid vaccine trials among soldiers who were deployed to India in 1904–1908. At that time, typhoid was a common disease across the Indian subcontinent, with high rates of morbidity and mortality in the preantimicrobial era [6]. Once chloramphenicol became widely available, typhoid case fatality rates fell dramatically (from as high as 30% to <1%), and it became a disease that could be easily managed [7]. Vaccination programs for typhoid (with the killed whole-cell typhoid-paratyphoid A-B vaccine), which had been initiated in the military, were widely implemented in India, but these programs were discontinued in the 1980s due to the reactogenicity of the vaccines. Most typhoid disease burden estimates in India originated from facilities where patients present with severe disease. However, the sustained problems of disease diagnosis, including low sensitivity of blood cultures and low specificity of serological tests, have hampered efforts to accurately measure the disease burden [4].

In the late 1970s and early 1980s, chloramphenicol resistance became widespread in India, but other effective antimicrobials such as cotrimoxazole were widely available [7]. Multidrug-resistant (MDR) *S.* Typhi (resistance to amoxicillin, chloramphenicol, and cotrimoxazole) emerged in the late 1980s and became widespread in the 1990s in India [8]. Fluoroquinolones, mainly ciprofloxacin, provided an alternative means of controlling the disease with even fewer complications and carriage than with older antimicrobials. Despite increasing quinolone resistance necessitating a switch to azithromycin, the proportion of *S*. Typhi isolates that demonstrated MDR has declined in recent years [9]. With rapid defervescence following the use of these newer-generation antimicrobials [10], the number of severe typhoid cases that required hospitalization also decreased. More recently, sentinel hospitals in India have been reporting fewer cases of typhoid; therefore, there has been a widespread perception that typhoid is no longer a problem [11]. However, an examination of limited data indicates that this is unlikely to be the case. Population-based cohort studies in the 1990s and early 2000s described a high disease incidence, particularly in children aged 2–4 years [12]. Widespread use of antimicrobials without prescription is well known across the Indian subcontinent [13] and may mask the diagnosis of the disease by both preempting formal healthcare-seeking and decreasing the sensitivity of blood culture [14]. The Severe Enteric Fever in India study, which combines hospital-based, laboratory-based, and active surveillance, has recently been initiated and will help us to better determine the full spectrum of illness and healthcare-seeking behavior for typhoid fever in India [15].

Similar to the spread of epidemic cholera [16], *S.* Typhi from Asia spread to Africa in the early 1990s, carrying multiple antimicrobial-resistant (AMR) genes. Detailed phylogenetic analyses of organisms using whole-genome sequencing demonstrated that the H58 subclade (now designated genotype 4.3.1) disseminated from India and other parts of South Asia in multiple seeding events and has since become the dominant genotype worldwide [17]. While this is a significant concern, still more disturbing information emerged from Pakistan in 2016. A new extensively drug-resistant (XDR) variant of *S*. Typhi was identified in Sindh, leaving azithromycin as the only effective oral therapy. This XDR variant carries multiple AMR genes, a number of which are harbored on a specific plasmid, and there is a fear that these could spread to other *S*. Typhi and *S*. Paratyphi A lineages [18]. Likewise, azithromycin resistance has been reported from other parts of Asia; if the Sindh strain acquires this additional resistance, few affordable treatment options remain (see Kirchhelle et al, S388 in this supplement). As community-based treatment becomes less effective at mitigating the symptoms of typhoid fever, we may be poised to see a resurgence of hospitalized cases across India.

## Typhoid in Asia: Nepal

Nepal is a country of more than 28 million people located in the Himalayan mountain range in South Asia. While never colonized, it served as a buffer between Imperial China and British India and underwent civil war in the 1990s and early 2000s. The documented history of typhoid fever and typhoid vaccines in Nepal begins around the late 1950s when school children received the whole-cell vaccine. Three decades later, one of the first landmark trials of the Vi-polysaccharide (ViPS) typhoid vaccine was conducted in Kathmandu, Nepal. The attack rate with typhoid fever was found to be 1620 cases per 100 000 person-years among the control group, documenting a substantial burden of disease in this population [19].

As a result of a letter exchange with Nepalese medical experts in the *New England Journal of Medicine* [20, 21], the author of a review article on typhoid fever, Dr Jeremy Farrar, decided to visit Nepal to personally observe the high rates of typhoid fever at Patan Hospital on the outskirts of Kathmandu. Subsequently, the Oxford University Clinical Research Unit based in Patan Hospital (OUCRU-Nepal) was established, commencing a decades-long research program to evaluate the diagnosis, treatment, and prevention of typhoid fever in Nepal. The first study that resulted from this collaboration was a case-series analysis of 609 consecutive typhoid fever cases, which revealed that typhoid and paratyphoid fevers were clinically indistinguishable [22]. Following this work, an era of collaborative randomized controlled trials (RCTs) on typhoid fever prevailed [23–26]. Access to whole-genome sequencing confirmed that H58 is the dominant *S.* Typhi clade in Nepal and described disease outcomes for this genotype, including the high rate of fluoroquinolone treatment failure [27]. Studies that identified the rates of gallbladder carriage of *S.* Typhi and Paratyphi A [28] and mathematical modeling of the drivers of trends in incidence [29] were also conducted.

While the ViPS vaccine was tested and licensed based on data from Nepal, predominantly only Western visitors made use of this vaccine until recently. OUCRU-Nepal facilitated a pilot ViPS vaccination project in the Lalitpur schools between 2010 and 2011. More recently, in 2018, more than 20 000 children in Lalitpur, Nepal, were vaccinated as part of an ongoing typhoid Vi-conjugate vaccine (TCV) RCT [30].

To address the more widespread lack of typhoid surveillance data in Nepal, the Surveillance of Enteric fever in Asia Project (SEAP) has been focusing on typhoid surveillance in urban and periurban areas east of Kathmandu [31]. These studies have identified an urgent need for a reliable, rapid diagnostic test that will help distinguish typhoid from other circulating organisms such as typhus, which causes a clinically indistinguishable disease that is often misdiagnosed. Such a test would enable more appropriate use of antimicrobial therapy that, in addition to vaccination, will help to control the rising threat of AMR.

## Typhoid in Asia: Vietnam

Vietnam is a highly populated (more than 95 million people as of 2017) and ecologically diverse tropical country in Southeast Asia. Of all the countries with a turbulent past in Southeast Asia, it is arguably Vietnam that has experienced the most economic and political upheaval in the latter part of the 20th century. After being on the verge of famine in the early 1980s, the developmental trajectory of Vietnam has since been truly astonishing; Vietnam has transformed from one of the least developed countries in Asia into a robust emerging economy. This transition was chiefly driven by an economic reform named Đổi Mới (“to make change”), which was put in place by the governing communist party in 1986 [32]. This policy has outperformed its projections, resulting in decreased poverty and an expansion of the middle classes. The consequences of development have been broad, but the population of Vietnam is now healthier and has a longer life expectancy. Between 1993 and 2012, infant and under-age-5 mortality rates decreased from 33 to 19 and from 45 to 24 per 1000 births per year, respectively [33]. Child stunting has also decreased over the same 20-year period, from 61% to 23%, and life expectancy has increased from 71 to 76 years.

Typhoid fever burden fundamentally epitomizes the success of the economic reforms in Vietnam and acts as a surrogate indicator of the effects of these reforms on the poorest sections of Vietnamese society [34]. Given that typhoid is largely a waterborne disease, improvements in infrastructure, education, and household living conditions have had a profound effect on the prevalence of the disease. In the early 1990s, when collection of reliable diagnostic data began, typhoid fever was rampant in Ho Chi Minh City [35]. Sustained epidemics were recorded and associated with infections that could not be treated with conventional first-line antimicrobials. The disease was not restricted to the major cities; it was highly endemic in the Mekong River Delta in the south and provinces bordering China in the north, with incidence rates >500 cases per 100 000 person-years [36, 37]. The government acknowledged the issue of typhoid fever and initiated various vaccination campaigns with the ViPS vaccine [37]. Eventually, this vaccine would be manufactured within Vietnam through the government public health system. Additionally, Vietnam became the location for the first clinical trial of a Vi-conjugate vaccine, which was found to provide prolonged immunity to disease [38].

Vietnam still has many persistent problems with respect to infectious diseases (including human immunodeficiency virus [HIV], dengue, and tuberculosis), but typhoid fever has largely vanished. Given the complexities of changes in Vietnamese society from the mid-1990s onward, it is difficult to disaggregate the direct effects of any particular intervention on the incidence of typhoid disease. Despite this limitation, the control of typhoid fever in Vietnam has been a public health triumph and one that other economically transitional countries can learn from. The economic reforms initiated by the government instigated a change in living standards for the vulnerable, provided improved sanitation for urban and rural populations, educated people in hygiene practices, and initiated an aggressive vaccination campaign. This combined strategy provides a blueprint for disease control and represents a case study of a country that can practically eliminate typhoid in a 10- to 15-year time frame.

## Typhoid in Oceania: Fiji

Fiji is an upper-middle-income Pacific island country (and former British colony) with a population approaching 900 000. Collaborations between public health authorities and international researchers have begun to investigate the recent and historical epidemiology of typhoid fever in Fiji. Despite typhoid case numbers in Oceania being modest in the context of global typhoid burden, modeled per capita incidence rates have been estimated to be high [2].

Against a backdrop of improving living standards, Fiji saw a sharp upturn in laboratory culture–confirmed typhoid fever cases between 2004 and 2008, from a small number of isolated cases annually to an incidence of approximately 400 cases per year (46 per 100 000 person-years), which has since declined [39]. Incidence rates in some provinces exceeded 100 per 100 000 person-years. The median age of cases was found to be 25 years (interquartile range, 15–36 years), which is higher than typically reported in typhoid-endemic countries. More than 90% of detected cases arise in indigenous iTaukei Fijians, who comprise just 57% of the population [40]. The majority of *S.* Typhi isolates are susceptible to first-line antimicrobials, and Fiji has a regulated antimicrobial stewardship program [40]. In 2010, Australian Aid supplied ViPS vaccines to high-incidence areas following Cyclone Tomas. The use of this vaccine in response to the outbreak reduced incidence in these settings, while incidence remained stable elsewhere in the island nation where typhoid vaccination is uncommon [41].

*S.* Typhi in Fiji form part of a Pacific clade that is thought to be genetically distinct from those circulating elsewhere. Introduction of the MDR H58 subclade to Fiji in the early 1990s was not sustained [17]. If *S.* Typhi is not new to Fiji, then what explains the recent upturn in numbers of typhoid fever cases? Serosurveillance (based on immunoglobulin G against the Vi-antigen) suggests that endemic transmission is more likely than a recent outbreak [42]. Seroprevalence increases by age, which is consistent with a long-standing force-of-infection that is stable across all age groups. The temporal rise in incidence may also be attributable to strengthened laboratory surveillance [43]. The observed peak incidence in young adults may be dependent on a combination of nonspecific disease presentations, lower sensitivity of blood culture in children (due to a low volume of blood drawn, and disinclination toward venepuncture of pediatric cases) [14]. With no difference reported in healthcare-seeking behavior, comparable seroprevalences in iTaukei and Indo-Fijians perhaps further suggests a role for human genetics in typhoid pathology [44].

The wet environment is a common thread in Fijian typhoid fever investigations. Risk factors for typhoid identified in a recent case-control study included unimproved or broken sewage systems, unwashed produce, intermittent water supplies (potentially substituted with surface water), and the absence of handwashing with soap [45]. Typhoid cases were also found to grow vegetables nearer to toilets and sewers than controls [46]. Seroprevalence was also higher in high rainfall and modeled flood-risk areas [47]. Hence, serology provides an important additional tool for understanding how the risk of typhoid fever varies over time and across different communities.

## Typhoid in Africa: Sierra Leone

Sierra Leone is a former British colony in tropical West Africa with a population of more than 7 million. Although accurate retrospective diagnosis is impossible, historical sources first mention typhoid fever in Sierra Leone in 1828 [48]. However, unlike in the other settings we describe, typhoid diagnostic capacity has remained largely unchanged since the colonial medical services first introduced the Widal agglutination test in the early 20th century. Annual reports from the Medical and Sanitary Department in Sierra Leone demonstrated how typhoid services were, under the colonial administration, organized primarily around the control of a disease with serious epidemic potential that might affect the health of European colonial staff. Reported numbers of cases remained low; however, the 1932 annual report remarked on “atypical” typhoid in Sierra Leone when 1 outbreak led to only 13 cases across the country, rather than a more serious outbreak as expected [49].

Typhoid disease trends changed following independence in 1961. During this period, there was a shift in perception from typhoid being primarily an epidemic to an endemic disease, resulting from a recognition of the substantial contribution typhoid made to the overall febrile disease burden in Sierra Leone (according to Widal tests) [50]. Despite ambitious proposals for a concerted investment in clinical and sanitation services, with a planned increase in the number of treatment centers from 34 to 1016, a period of single-party rule followed by a major economic depression led to deterioration of healthcare services by the 1980s. This had a knock-on effect on typhoid reporting, with only a single case reported to the Endemic Diseases Control Unit in 1980 [51]. Diagnostic, treatment, and surveillance services deteriorated even further during the civil war from 1991 to 2002.

Presently, the diagnosis and treatment of typhoid fever in Sierra Leone faces a number of challenges. The laboratory facilities that are necessary for blood-culture confirmation are lacking, and other tests are excluded from the government’s Basic Package of Essential Health Services [52]. As free supplies of antimicrobials are frequently out of stock, out-of-pocket costs for typhoid treatment add to the financial barrier to effective typhoid management. Despite their low specificity and loose interpretability, Widal tests remain one of the few opportunities available to clinicians to provide a definitive diagnosis for febrile illnesses. This limitation holds important implications for both the professional identity and livelihoods of these clinicians.

From a public health perspective, typhoid surveillance has benefited from recent major investment in a revitalized Integrated Disease Surveillance and Response (IDSR) program, following the 2014–2016 Ebola epidemic. More than 75 000 suspected cases of typhoid were reported through IDSR in 2016 [53], the majority of which were diagnosed based on clinical signs and symptoms only. Despite relying on more or less the same diagnostic technology for nearly a century, this increase in reported typhoid cases (from a single case in 1980) likely reflects changes in socioeconomic conditions, demographics, healthcare access, and clinical case definitions rather than actual changes in incidence. The large variability in reported case numbers makes it difficult to estimate the current burden of typhoid disease with any certainty, which has important implications for assessing the cost-effectiveness of a vaccine or other public health program in the country. Consistent, reliable, and low-cost surveillance tools, along with models, are therefore needed to estimate the burden of typhoid fever with any confidence.

## Typhoid in Africa: Malawi

Malawi is a small, subtropical country in southeastern Africa with a population of more than 18 million. It was a British protectorate until it gained independence in 1964 and is currently one of the poorest countries in the world. Yet, through the work of the Malawi-Liverpool Wellcome Clinical Research Programme (MLW), it has one of the richest longitudinal bacteremia datasets from a single healthcare facility on the continent [54]. Prior to 2011, between 10 and 20 cases of *S.* Typhi were isolated from patients in Queen Elizabeth Central Hospital (QECH) in Blantyre annually [55]. During that time, as was the case across much of sub-Saharan Africa, the dominant pathogen grown from blood cultures was *Salmonella enterica* serovar Typhimurium (*S.* Typhimurium), a nontyphoidal serovar associated with the dominant risk factors of malaria, malnutrition, and immunosuppression secondary to advanced HIV infection [56, 57].

Beginning in 2011, an epidemic increase in cases of *S.* Typhi was observed at QECH, peaking in 2013 with 843 cases [58]. Whole-genome sequencing was used to explore this rise and revealed multiple introductions of MDR H58 into Malawi and other parts of east and southern Africa from the Asian subcontinent [17]. At that time, fluoroquinolones were not widely available at community health centers and pharmacies. Mathematical modeling was able to explain the observed epidemic based on surveillance data from QECH and predicted that the number of cases would decrease following the epidemic peak in 2013–2014 but stabilize at an endemic level that was approximately 10-fold higher than prior to the epidemic [59]. Unpublished data from the Strategic Typhoid Alliance across Africa and Asia has confirmed this ongoing high incidence of disease from a single urban township in Blantyre [60]. Meanwhile, additional typhoid outbreaks have been reported from numerous locations throughout the country, including 1 outbreak with uncharacteristically high levels of neurological sequalae [61].

Typhoid cases occur predominantly among school-age children in Malawi, with disease identified in the first year of life and peaking in the 5- to 9-year age group [55], suggesting that routine vaccination of infants, including a catch-up campaign that targets children aged ≤15 years, may be an effective strategy for controlling the disease. Notably, unlike other forms of *Salmonella*, there does not appear to be an association with HIV infection or malaria; *S.* Typhi is isolated throughout the year, with a peak during the rainy, hotter months of December through May, peaking in April [58].

Due to the research infrastructure within the country and longitudinal data generated by MLW on disease burden, Malawi is the first country in Africa to host an efficacy trial for the newly World Health Organization (WHO)–prequalified typhoid conjugate vaccine, Tybar-TCV, through the TyVAC Consortium [30]. Following from this, and based on recommendations from the WHO Strategic Advisory Group of Experts (SAGE) and the provision of Gavi funding [62, 63], it is hoped that Malawi will be one of the first countries in Africa to introduce TCVs into the extended program for immunization, reducing disease burden and protecting children across the country.

## DISCUSSION 

In the early part of the 21st century, typhoid is still a major global health issue but is largely unrecognized. This lack of recognition is due to multiple factors (Table 1). First, the burden of typhoid tends to be greatest in poor communities where the facilities necessary for appropriate diagnosis of the disease are lacking, as is the case in Sierra Leone and many other sub-Saharan African countries. Second, antimicrobials are readily available in the community in many countries where the disease incidence is high, such as in India, Nepal, and other parts of south and southeast Asia. While antimicrobials are capable of mitigating the more severe symptoms of typhoid fever, they also likely reduce formal healthcare-seeking and interfere with confirmatory diagnoses [14]. Last, even in locations with good surveillance, the incidence of culture-confirmed typhoid fever can change rapidly, particularly following the emergence of novel AMR variants, as occurred in Malawi [58] and other eastern African countries [64–67]. The apparent opposing trajectories of typhoid fever incidence in Vietnam and Fiji, both of which have undergone recent improvements in the overall standard of living and implemented vaccination campaigns targeted at typhoid fever [37, 40], highlight the importance of comprehensive and consistent surveillance to understand the factors that drive complex disease dynamics.

Partnerships among local clinicians, public health officials, and international researchers have helped to develop, strengthen, and sustain the laboratory infrastructure necessary to maintain typhoid fever surveillance across Africa and Asia. Additionally, these interactions have led to pivotal RCTs and observational studies that have informed treatment and prevention strategies and will continue into the future. Notably, local researchers in LMICs have led the majority of these studies. Further developing the capacity to recognize, treat, and prevent typhoid fever in these locations will become even more critical as we move into a period of typhoid elimination. The future of typhoid fever surveillance and control relies on promotion of the independence and local ownership of research and policy decisions made in endemic countries.

We are at an important crossroad for typhoid fever control. The recent WHO prequalification and SAGE recommendation for the routine use of typhoid conjugate vaccine provides a cost-effective means of controlling typhoid fever in endemic countries [60], but political will is needed to ensure that TCV strategies are implemented. Decisions made now may influence whether typhoid fever is eventually eliminated as a public health problem or whether the threat of AMR *S.* Typhi, including the emergence of XDR *S.* Typhi in 2016 [18], threatens to return us to an era of relatively ineffective antimicrobial treatment and high morbidity and mortality.

## Notes

***Financial support.*** This work was supported by funding from the Bill & Melinda Gates Foundation (grants OPP1116967, OPP1151153), the Wellcome Trust (Strategic Award 106158/Z/14/Z, grant 102919/Z/13/Z to F. P. M.), and the Medical Research Council (grant MR/J003999/1 to C. H. W.). This work was also supported by grants from the World Health Organization–Western Pacific Region and Chadwick Trust (to C. H. W.).

***Supplement sponsorship.*** This supplement has been funded by the New Venture Fund.

***Potential conflicts of interest.*** V. E. P. has received reimbursement from Merck for travel expenses unrelated to the subject of this article and is a member of the World Health Organization Immunization and Vaccine-related Implementation Research Advisory Committee. All other authors report no potential conflicts of interest. All authors have submitted the ICMJE Form for Disclosure of Potential Conflicts of Interest. Conflicts that the editors consider relevant to the content of the manuscript have been disclosed.
